# Right colectomy from open to robotic — a single-center experience with functional outcomes in a learning-curve setting

**DOI:** 10.1007/s00423-022-02576-8

**Published:** 2022-06-09

**Authors:** Markus Hirschburger, Rolf Schneider, Sophie Kraenzlein, Winfried Padberg, Andreas Hecker, Martin Reichert

**Affiliations:** 1 Department of General, Visceral and Thoracic Surgery, Hospital of Worms, Gabriel-von-Seidl-Strasse 81, 67550 Worms, Germany; 2 Department of General, Visceral, Thoracic, Transplant and Pediatric Surgery, University Hospital of Giessen, Rudolf-Buchheim-Strasse 7, 35390 Giessen, Germany

**Keywords:** Right colectomy, Complete mesocolic excision, Robot, Intracorporal anastomosis, Bowel dysfunction, Ileus

## Abstract

**Purpose:**

Right colectomy (RC) is a frequently performed procedure. Beneath standard conventional open surgery (COS), various minimally invasive techniques had been introduced. Several advantages had recently been described for robotic approaches over COS or conventional laparoscopy. Nevertheless, novel minimally invasive techniques require continuous benchmarking against standard COS to gain maximum patient safety. Bowel dysfunction is a frequent problem after RC. Together with general complication rates postoperative bowel recovery are used as surrogate parameters for postoperative patient outcome in this study.

**Methods:**

Retrospective, 10-year single-center analysis of consecutive patients who underwent sequentially either COS (*n* = 22), robotic-assisted (ECA: *n* = 39), or total robotic surgery (ICA: *n* = 56) for oncologic RC was performed.

**Results:**

The conversion from robotic to open surgery rate was low (overall: 3.2%). Slightly longer duration of surgery had been observed during the early phase after introduction of the robotic program to RC (ECA versus COS, *p* = 0.044), but not anymore thereafter (versus ICA). No differences were observed in oncologic parameters including rates of tumor-negative margins, lymph node-positive patients, and lymph node yield during mesocolic excision. Both robotic approaches are beneficial regarding postoperative complication rates, especially wound infections, and shorter length of in-hospital stay compared with COS. The duration until first postoperative stool is the shortest after ICA (COS: 4 [2–8] days, ECA: 3 [1–6] days, ICA: 3 [1–5] days, *p* = 0.0004). Regression analyses reveal neither a longer duration of surgery nor the extent of mesocolic excision, but the degree of minimally invasiveness and postoperative systemic inflammation contribute to postoperative bowel dysfunction, which prolongs postoperative in-hospital stay significantly.

**Conclusion:**

The current study reflects the institutional learning curve of oncologic RC during implementation of robotic surgery from robotic-assisted to total robotic approach without compromises in oncologic results and patient safety. However, the total robotic approach is beneficial regarding postoperative bowel recovery and general patient outcome.

## Introduction

Right colectomy (RC) with complete mesocolic excision (CME) for carcinoma of the ascending colon is a frequently performed standard procedure in general surgery. Several different techniques are known for oncologic RC with CME, ranging from conventional open surgery (COS) to hybrid or total minimally invasive approaches with different variants of extracorporal or intracorporal techniques for the ileo-colic anastomosis, respectively [1–3]. Thereby, numerous studies have proven the non-inferiority of minimally invasive surgery in colorectal cancer concerning oncological long-term results compared with COS as the benchmark [4–7]. Although conventional laparoscopy had broadly been applicated as standard in surgery of the left colon and rectum over the last decades, minimally invasive approaches using conventional laparoscopy are sparsely introduced into clinical routine practice for oncologic resections of the right colon [8–11]. However, through increasing numbers and availability of robotic programs, growing experiences with, further development in as well as technical advantages of robotic-assisted surgery over conventional laparoscopy [2, 8, 12–16], minimally invasive robotic approaches for RC are constantly evolving and progressively established in the clinical routine [9, 11, 16–18]. Although costs of minimally invasive approaches, especially of robotic surgery, are considerably higher compared with COS [11, 17, 19, 20], some evidences exist in the current literature concerning improved postoperative short-term outcome and morbidity, respectively, of patients after minimally invasive RC with CME [11, 17, 18, 20–24]. Particularly the total minimally invasive approach with intracorporal anastomosis is beneficial considering postoperative pain [25], surgical site infections [22, 26], and complications urging re-interventions [22, 26, 27] and finally results overall in a shorter recovery and decreased postoperative length of hospital stay [22, 25–29]. After this had been extensively shown in studies and confirmed by systematic reviews and metaanalyses for conventional laparoscopic apporaches [30], current literature reveals further improvements of intraoperative visualization resulting in higher lymph node yield and postoperative patient outcome with growing experiences in minimally invasive robotic surgery for RC with CME [8, 24, 30, 31]. Nevertheless, the beneficial impact of robotic-assisted or total robotic surgery in comparison with COS as the benchmark approach for oncologic RC with CME on postoperative outcome, especially including systemic inflammation, functional bowel recovery, and their association with duration of hospitalization, currently remains elusively. While postoperative bowel dysmotility and paralysis is a temporary physiologic reaction after abdominal surgery, a prolonged state of bowel dysfunction and paralytic ileus is an extraordinary frequent clinical problem in patients after surgery of the right hemicolon [24, 32]. Nonetheless, their pathophysiology is currently not fully understood, but delayed postoperative return to normal bowel function is a major threat for patients, which frequently results in high risk for harmful acid aspiration, impaired patient comfort, and general postoperative outcome and prolongs hospital stay as well as recovery of affected patients and consequently increases public health costs [33–35].

We herein report the experiences with oncologic RC including CME from a nation-wide benchmark center for robotic colorectal surgery [36] with regard to postoperative bowel recovery and patient outcome. Before implementation of the robotic program at that institution, RC was approached by COS. During the learning curve of robotic surgery, the complex procedure of oncologic RC with CME was initially performed by a robotic-assisted approach with extracorporal ileo-colic anastomosis and was finally converted to total minimally invasive robotic surgery with intracorporal anastomosis, giving the opportunity to compare both minimally invasive approaches with standard COS in the era of robotic surgery.

## Material and methods

### Patients

This retrospective single-center cohort study was formally approved by the local ethics committee of the Landesaerztekammer Rheinland-Pfalz (approval No. 2019–14732-retrospektiv). The study was performed in accordance with the latest version of the Declaration of Helsinki. The data are collected, and the manuscript is written and submitted in accordance with the COPE guidelines. All patients were treated according to the institutional standard of care.

All consecutive 117 patients who underwent elective oncological RC with CME due to preoperative concerns or histologically proven malignancy were included into the data analysis from 01/2013 to 03/2022. Through implementation of a robotic surgery program at the institution, the surgical technique was converted from a conventional open surgical approach (COS: 01/2013–12/2014, *n* = 22 patients) to minimally invasive robotic surgery as clinical standard for oncological RC with CME during the 10-year observational period. Thereby the robotic approach by itself underwent some evolution from robotic-assisted RC with extracorporal (ECA: 01/2015–12/2017, *n* = 39 patients) to total minimally invasive robotic technique with intracorporal hand-sewn ileo-colic anastomosis (ICA: 01/2018–03/2022, *n* = 56 patients).

Patient data were analyzed retrospectively from the prospectively maintained institutional database regarding general patient characteristics and surgical procedure characteristics, general postoperative patient outcome, and more specifically regarding postoperative day of first stool as the surrogate parameter for postoperative bowel recovery or dysfunction. Perioperative peripheral blood leukocyte counts and C-reactive protein (CRP) values were obtained from routine laboratory examinations. The highest values of leukocytes and CRP on POD 1–3 were used as markers for interpreting surgical trauma-induced systemic inflammation.

### Surgery and perioperative patient care

All patients received mechanical bowel preparation at the day before surgery and antibiotic single-shot prophylaxis with ceftriaxone (2 g iv) and metronidazole (500 mg iv) immediately before surgery. Median laparotomy was used for COS. Mobilization of the right colon and CME were approached by a lateral-to-medial approach with central ligation of the ileo-colic vessels and consecutive dissection of the lymph nodes across the CME plain. Transverse colon and ileum were transected by a stapling device and the bowel continuity was restored by side-to-side ileo-colic hand-sewn running suture anastomosis.

**Fig. 1:**
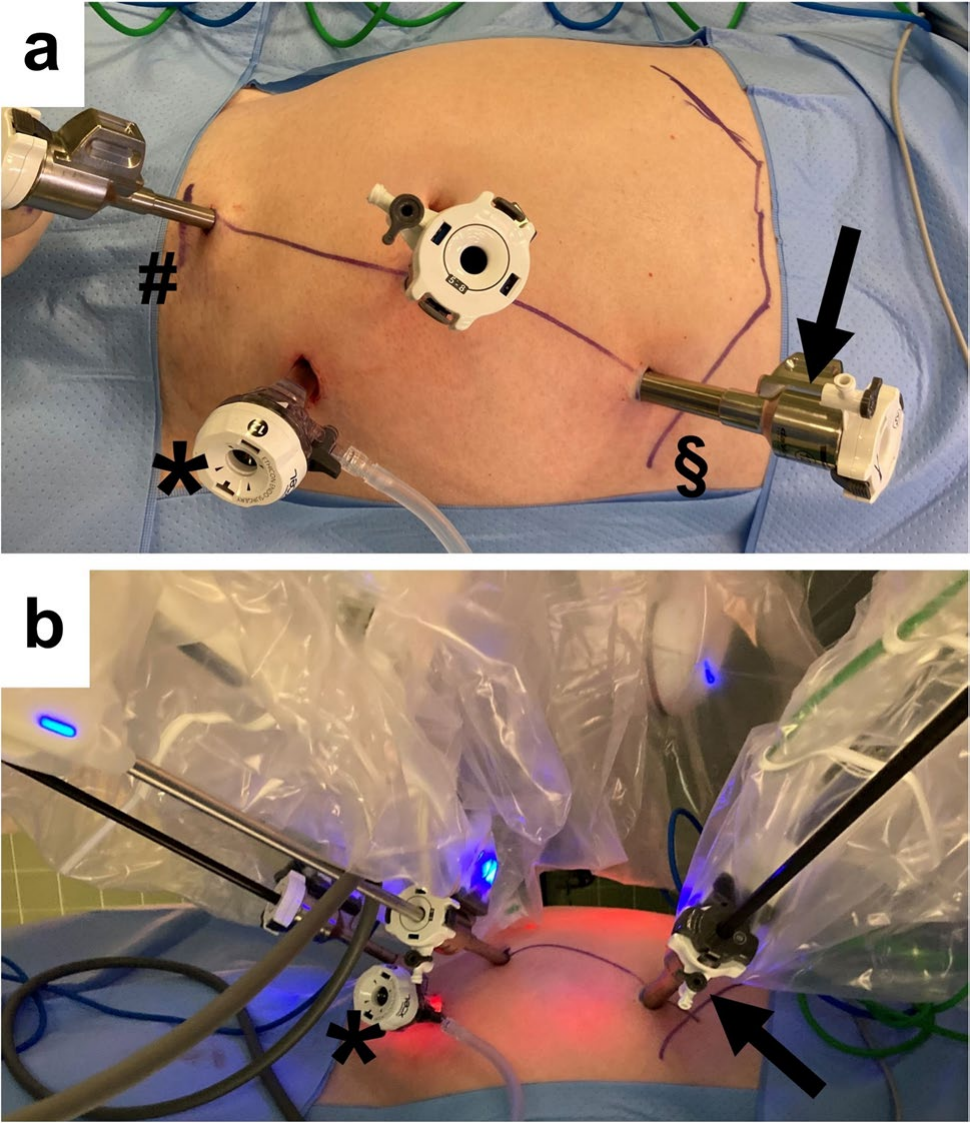
Port placement for right colectomy with the Da Vinci ® Xi system (Intuitive Surgical, Sunnyvale, CA, USA). Three robotic ports and one assistant port (*) were used. **a** The 12-mm assistant port (*) is placed in the left lower abdomen by means of a minilaparotomy. Thereafter, the three robotic ports are introduced under vision on a line between the symphysis (#) and the left costal arch (§) on the midclavicular line (two 8-mm ports and one 12-mm port [→]). Care has to be taken on the assistant port, which should be placed in a triangle between the two lower robotic ports to have optimal access. For the later Pfannenstiel minilaparotomy the access of the lowest robotic port above the symphysis is used. **b** The lowest robotic port above the symphysis is used for bipolar instrument. The middle robotic port is used for the camera and the upper port in the left upper abdomen beneath the costal arch (→) is used for monopolar scissors, stapler devices, and in case of intracorporal anastomosis for the needle holder

For robotic procedures the Da Vinci Si system was used from 01/2015 to 12/2017. From 01/2018 all procedures were performed with the Da Vinci Xi system (Intuitive Surgical, Sunnyvale, CA, USA). As shown in Fig. 1a, three-port robotic technique with one assistant port was used for surgery of the right colon. The 12-mm “assistant” port was introduced through a minilaparotomy in the left lower abdomen. After establishing the pneumoperitoneum at 15 mm Hg three “robotic” ports (two 8 mm and one 12 mm) were placed in the left midclavicular line between the symphysis and the costal arch under vision. Surgery was performed in slight Trendelenburg position (~ 10–15°) to the left (~ 10°). Robotic oncologic RC followed the principles of COS. The CME was performed by a medial-to-lateral approach with central ligation of the ileo-colic vessels and lymph node dissection. Transverse colon and ileum were transected by stapling devices. In the ECA group the specimen was retrieved and the ileo-colic anastomosis was performed by the COS technique through a 7–10-cm transverse laparotomy in the right upper abdomen. In the ICA group, ileum and transverse colon were positioned side-to-side. Ileum and colon were opened by an incision with the robotic monopolar scissors and the side-to-side ileo-colic anastomosis was performed by bi-directional running suture with Stratafix™ (3/0). Finally, the specimen was retrieved through a 5-cm Pfannenstiel incision. At the end of the procedure the gastric tube was removed and the patients started drinking on the day of surgery and were on liquid diet until the first postoperative day.

### Statistical analysis

The patient cohort was subdivided into three groups regarding the surgical approach: (1) conventional open surgery (COS group, *n* = 22 patients), (2) the either group of patients who underwent a robotic-assisted, minimally invasive approach with extracorporal anastomosis (ECA group, *n* = 39 patients), and (3) the other group of patients who underwent a total minimally invasive, robotic approach with intracorporal anastomosis (ICA group, *n* = 56 patients).

Statistical analyses were performed using GraphPad Prism (Version 9 for Windows, GraphPad Software, San Diego, CA, USA; www.graphpad.com). For descriptive statistics, categorical data were analyzed using Fishers exact test or Pearson’s *χ*^2^ test. Group comparisons of continuous variables were performed by Kruskal–Wallis test for global effects and, if applicable (i.e. *p*_*KW* _≤ 0.05), followed by Dunn’s test (corrected for multiple comparisons) for pairwise multiple comparisons.

Simple linear regression analysis was used to predict significant dependencies between relevant variables.

Data are given in median and minimum to maximum ranges for continuous variables as well as *n* (%) for categorical data. Bars in the boxplots, shown in the figures, depict medians. Whiskers indicate minimum to maximum ranges. The boxes extend from the 25th to 75th percentiles and indicate the interquartile ranges.

The *p*-values ≤ 0.05 were considered to indicate statistical significance. Because of the exploratory character of the study no adjustments of *p*-values were performed.

## Results

### Basic characteristics of patients and procedures

Basic patient characteristics and relevant chronic diseases were widely balanced between the three groups of patients (Table 1). The overall conversion rate in the robotic cohort from an initially intended robotic approach to COS for RC was low (3.2%). Two patients from the ECA group and one patient from the ICA group underwent conversion to COS due to dense adhesions, bleeding, and unclear definition of the tumor site during minimally invasive surgery, respectively (ECA: 5.1% and ICA: 1.8%, *p* = 0.566). These patients, who underwent conversion to open surgery, were excluded from further (postoperative) outcome analyses.

**Table 1:** Patient characteristics

Variable	Conventional open surgery (*n* = 22)	Robotic surgery		*p*-Value
		ECA (*n* = 39)	ICA (*n* = 56)	
Male gender (*n patients*)	14	19	25	0.317
Age (*years*)	73 (44–89)	71 (39–87)	74 (41–90)	0.268
BMI (*kg/m*^*2*^)	26.7 (20.7–41.1)	26.8 (17.2–39.5)	25.1 (16.7–39.5)	0.167
ASA (*score*)	2 (2–3)	2 (1–3)	2 (1–4)	0.165
Chronic diseases (*n patients*)	22	32	47	0.113
Arterial hypertension	18	24	37	0.254
Coronary artery disease	2	4	11	0.320
Chronic pulmonal disease	2	2	11	0.097
Diabetes mellitus	3	4	7	0.913
Chronic kidney disease	4	5	2	0.093
Systemic immunosuppression	0	4	7	0.229
Neurologic disease	2	6	10	0.627
Previous malignoma (*n patients*)	6	6	10	0.505
Active smoking (*n patients*)	5	9	6	0.214
Active alcohol abuse (*n patients*)	0	0	1	0.577
Previous abdominal surgery (*n patients*)	11	13	24	0.414

*BMI* body mass index, *ASA* American Society of Anaesthesiologists physical status classification, *ECA* hybrid minimally invasive, robotic-assisted right colectomy with extracorporal hand-sewn anastomosis, *ICA *total minimally invasive, robotic right colectomy with intracorporal hand-sewn anastomosis

**Fig. 2:**
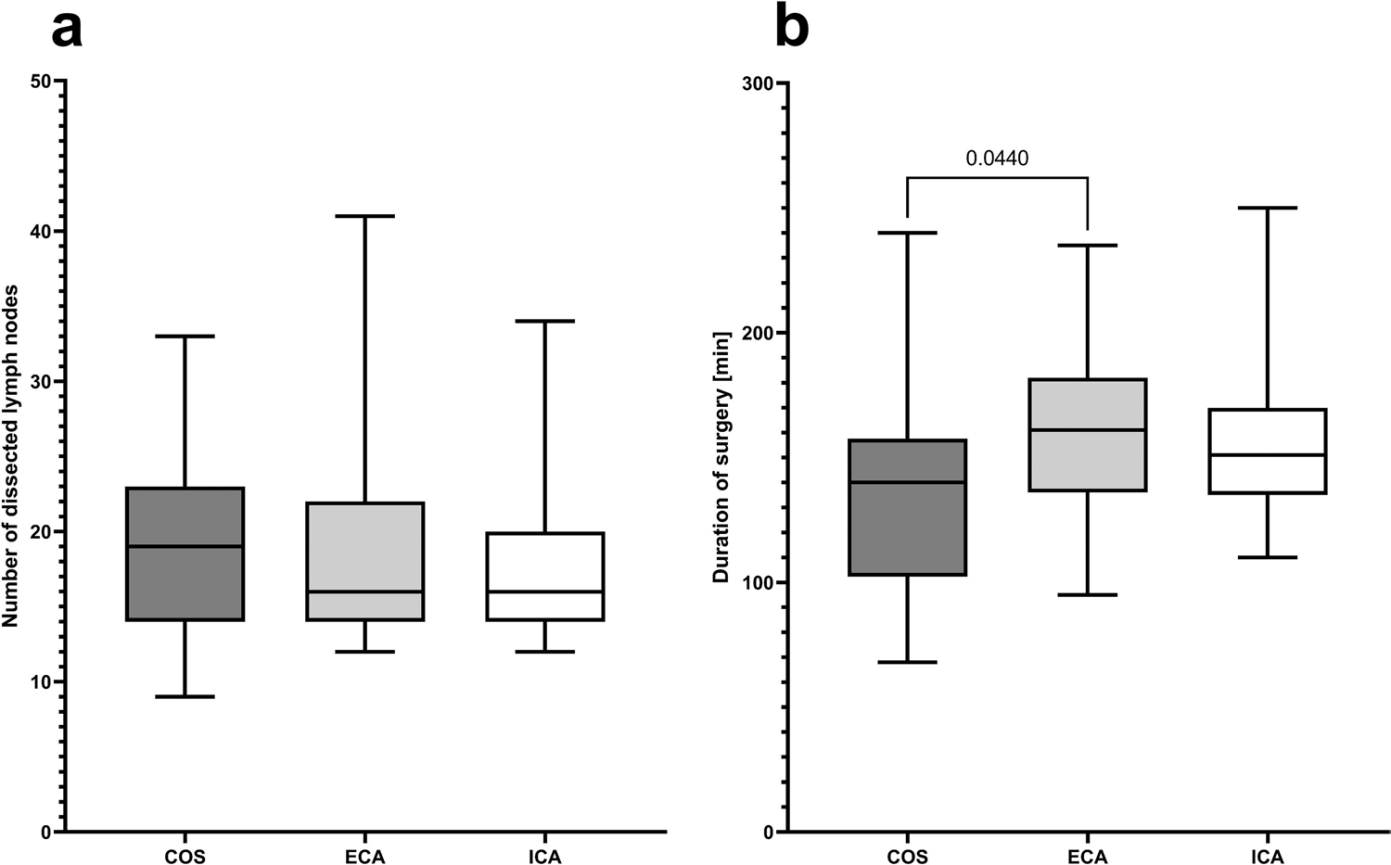
Surrogate outcomes of the surgical procedure. **a** Numbers of harvested lymph nodes during oncological right colectomy with complete mesocolic excision (*p* [Kruskal–Wallis test] = 0.570) and **b** duration of surgery (*p* [Kruskal–Wallis test] = 0.047). *COS* conventional open surgery; *ECA* hybrid minimally invasive, robotic-assisted right colectomy with extracorporal hand-sewn anastomosis; *ICA* total minimally invasive, robotic right colectomy with intracorporal hand-sewn anastomosis

Although all procedures were intended as oncological RC with complete mesocolic excision due to preoperative concerns or histologically proven malignancy, colon carcinoma was postoperatively histopathologically confirmed in 18 (81.8%), 22 (59.5%), and 47 (85.5%) of the cases from the COS, ECA, and ICA groups, respectively (*p* = 0.055). However, no differences were observed regarding tumor sizes (T-factor), rates of tumor-negative resection margins (R-factor), rates of nodal positive patients (N-factor), and the numbers of retrieved lymph nodes between the groups as important parameters for radical oncologic surgery (Table 2; Fig. 2). Notably, the duration of surgery was only slightly longer compared with COS during the ECA phase immediately after introduction of the robotic program for RC (COS: 140 [68–240] min versus ECA: 161 [95–235] min, *p* = 0.044). This effect was abandoned later on in ICA patients (151 [110–250] min, *p* = 0.154 versus COS; Fig. 2).

**Table 2:** Perioperative results and outcome

Variable	Conventional open surgery (*n* = 22)	Robotic surgery		*p*-Value
		ECA (*n* = 39)	ICA (*n* = 56)	
Final diagnosis^*^				
Divertikulitis	0	2	1	0.055
Large adenoma	4	13	7	
Carcinoma	18	22	47	
pT 1	3	4	13	0.785
pT 2	2	6	9	
pT 3	11	10	21	
pT 4	2	2	4	
pN +	5	4	9	0.704
Local pR0	All patients	All patients	All patients	1
Adhesiolysis	10	15	18	0.528
Intraoperative complications	1^µ^	1^µ^	0	0.333
Intraoperative conversion^#^	-	2	1	0.566
Intraoperative transfusion	1	1	1	0.786
Intraoperative drainage	5	2	0	0.0007
Postoperative complications (*n* patients)^¶^	13	6	5	< 0.0001
Surgical site infections	6	2^§^	0	< 0.0001
Pneumonia	2	0	1	
Intraluminal bleeding	1	0	3	
Intraabdominal bleeding	1	1^£^	0	
Anastomotic leakage	2^&^	1	1^£^	0.262
Internal hernia	1^¥^	0	0	
Abdominal re-do surgery	2	2	1	
Other	1	1	0	
CCI	8.7 (0–34.8)	0 (0–42.4)	0 (0–100)	< 0.0001
Grade I (*n* complications)^€^	6	2	0	
Grade II (*n* complications)^€^	6	2	1	
Grade IIIa (*n* complications)^€^	0	0	3	
Grade IIIb (*n* complications)^€^	2	2	1	
Grade IVa (*n* complications)^€^	0	1	0	
Grade IVb (*n* complications)^€^	0	0	0	
Mortality	0	0	2	*p* = 0.336
Postoperative return to ICU (*n* patients)	2^Ω^	1^ΩΩ^	3^ΩΩΩ^	0.537
Postoperative bowl stimulation (*n* patients)	7	12	21	0.553
Neostigmin	0	1	0	
Laxantives	4	5	2	
Movicol	1	7	16	
Klysma	6	2	2	

Patients who underwent conversion from an initially intended minimally invasive approach to conventional open surgery (*n* = 3) were excluded from perioperative outcome analysis. All procedures were performed due to preoperative concerns or histologically proven malignancy

^*^The “final” histopathologically confirmed diagnosis; patients who underwent intraoperative conversion to open surgery were excluded

^#^Overall conversion rate was 3.2%

^µ^Bleeding in both cases; in the ECA group leading to conversion to open surgery. Reasons for conversion to open surgery from an initially intended minimally invasive approach were dense adhesions and bleeding in the ECA group and unclear definition of the tumor site in the ICA group

^¶^Postoperative complications during the postoperative day 30 were included

^§^One deep organ space surgical site urging re-do surgery

^&^One anastomotic insufficiency urging re-do surgery. The other anastomotic insufficiency in the COS group as well was the anastomotic insufficiency from the ECA group were covered and did not require re-interventional therapy

^£^Urging re-do surgery

^¥^Internal hernia causing ileus and urging re-do surgery

^€^Regarding the Clavien-Dindo classification of surgical complications [60]. Ω Due to re-do surgery in both cases, Ω Ω due to acute kidney injury and Ω Ω Ω due to pneumonia (*n* = 1), intraluminal bleeding (*n* = 1), and intraluminal bleeding at postoperative day 6 and severe prolonged sepsis after re-do surgery for anastomotic leakage (*n* = 1). *CCI* comprehensive complication index [61], *ICU* intensive care unit, *ECA *hybrid minimally invasive, robotic-assisted right colectomy with extracorporal hand-sewn anastomosis, *ICA *total minimally invasive, robotic right colectomy with intracorporal hand-sewn anastomosis

### Perioperative C-reactive protein and leukocytes

**Fig. 3:**
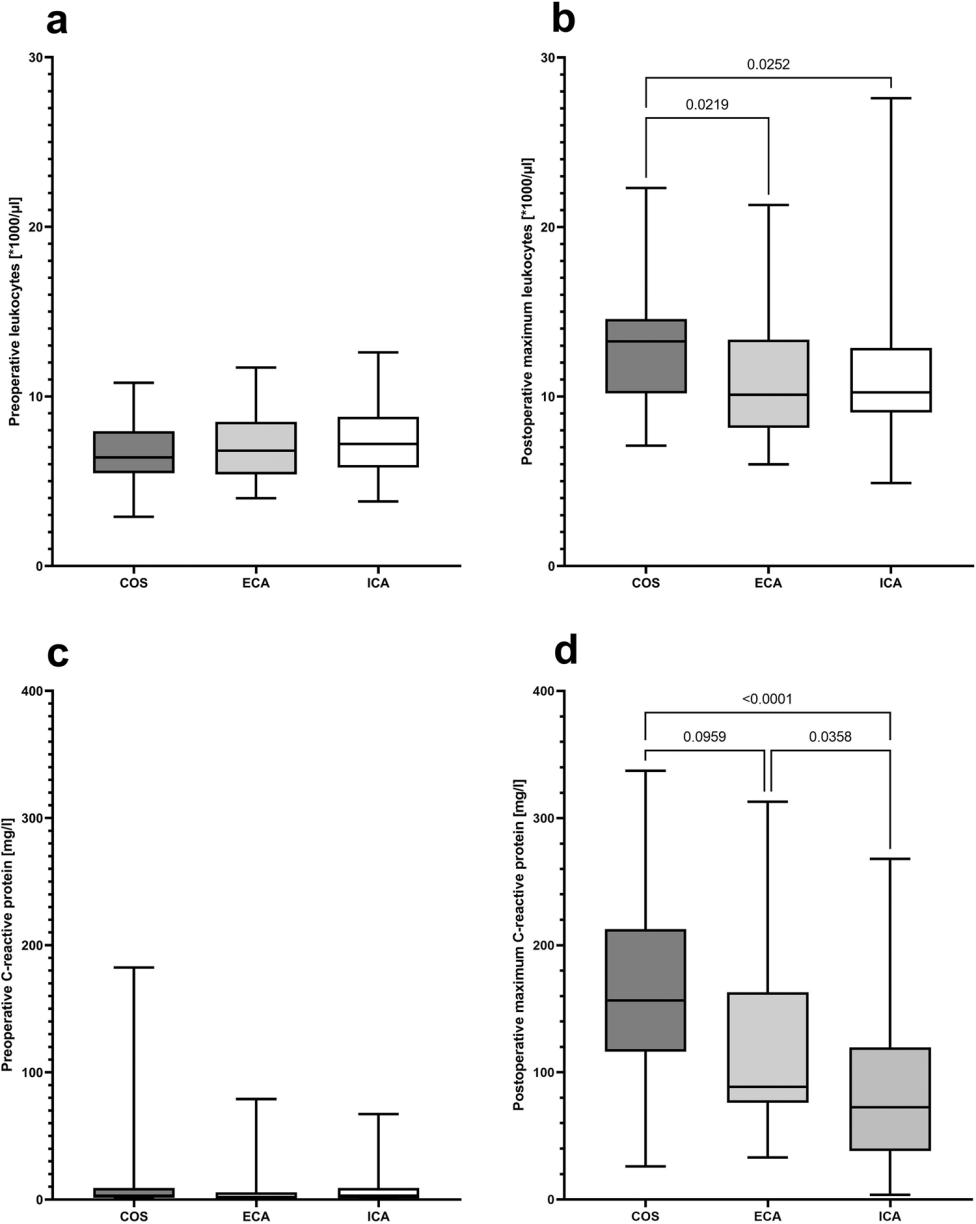
Perioperative markers for systemic inflammation. **a** Preoperative leukocyte counts in peripheral blood (*p* [Kruskal–Wallis test] = 0.411) and **b** highest leukocyte counts in peripheral blood during postoperative days 1–3 (*p* [Kruskal–Wallis test] = 0.014). **c** Preoperative C-reactive protein values in peripheral blood (*p* [Kruskal–Wallis test] = 0.408) and **d** highest C-reactive protein values in peripheral blood during postoperative days 1–3 (*p* [Kruskal–Wallis test]  < 0.0001). *COS* conventional open surgery; *ECA* hybrid minimally invasive, robotic-assisted right colectomy with extracorporal hand-sewn anastomosis; *ICA* total minimally invasive, robotic right colectomy with intracorporal hand-sewn anastomosis

The highest postoperative CRP values and leukocyte counts during POD 1–3 were used to estimate the extent of surgical trauma. No differences were observed between the three groups in preoperative CRP values and leukocyte counts; however, these markers of systemic inflammation were the highest postoperatively in the group of patients who underwent primary conventional open RC (Fig. 3).

### Postoperative outcome and recovery of bowel function

**Fig. 4:**
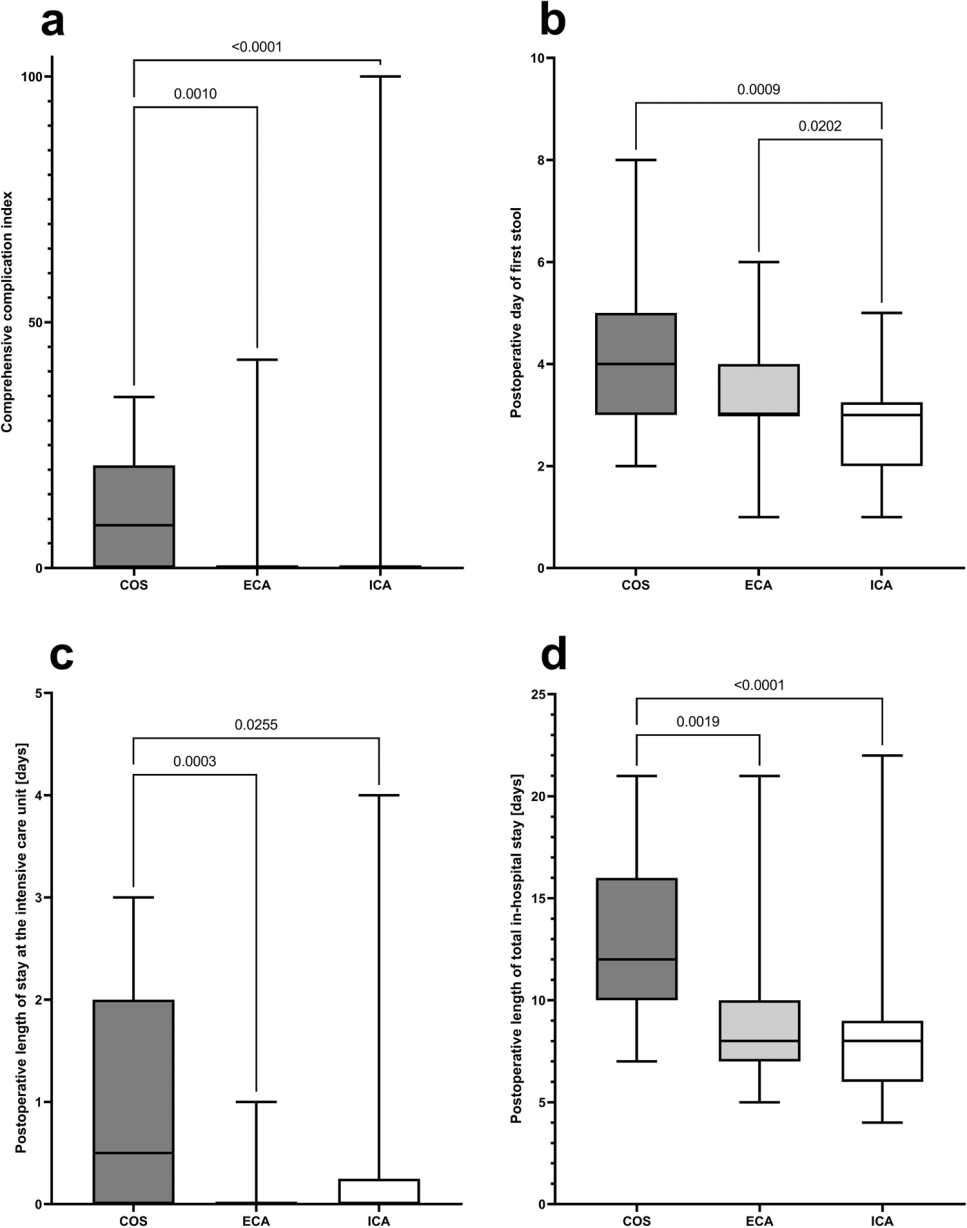
Surrogate parameters of postoperative patient outcome. **a** Postoperative comprehensive complication index summarizes postoperative complications classified by the Clavien-Dindo classification of surgical complications [60, 61] (*p* [Kruskal–Wallis test] < 0.0001). **b** Postoperative day of first stool (*p* [Kruskal–Wallis test] = 0.0004). **c** Initial postoperative length of stay at the intensive care unit (*p* [Kruskal–Wallis test] = 0.0006). **d** Postoperative length of total in-hospital stay (*p* [Kruskal–Wallis test] < 0.0001). *COS* conventional open surgery; *ECA* hybrid minimally invasive, robotic-assisted right colectomy with extracorporal hand-sewn anastomosis; *ICA* total minimally invasive, robotic right colectomy with intracorporal hand-sewn anastomosis

More patients from the COS group suffered from any postoperative complication, resulting in the highest ranks of comprehensive complication indexes in this group of patients (Fig. 4). Especially the rate of surgical site infections was the highest in the open RC group (COS: 27.3% vs. ECA: 5.4% vs. ICA: 0, *p* < 0.0001; Table 2). The duration until first postoperative defecation was used as the surrogate parameter for postoperative bowel recovery or dysfunction after RC. Although no differences were observed in the rates of postoperative bowel stimulation among the groups, duration until postoperative first stool was the shortest in the ICA group (COS: 4 [2–8] days vs. ECA: 3 [1–6] days vs. ICA: 3 [1–5] days, *p*_KW_ = 0.0004). Longer duration of postoperative bowel recovery and higher rates of postoperative complications as well as higher ranks of comprehensive complication indexes in the COS group resulted in longer postoperative stay at intensive care unit (COS: 0.5 [0–3] days vs. ECA: 0 [0–1] day vs. ICA: 0 [0–4] days, *p*_KW_ = 0.0006) as well as longer postoperative total in-hospital stay of these patients (COS: 12 [7–21] days vs. ECA: 8 [5–21] days vs. ICA: 8 [4–22] days, *p*_KW_ < 0.0001) (Table 2; Fig. 4). However, we complained two patients from the ICA group, who suffered from postoperative mortality (overall mortality rate in the study cohort: 1.7%, *p* = 0.336 for COS [0%] versus ECA [0%] versus ICA [3.6%]): one patient died on postoperative day 3 after ICA due to severe pulmonary embolism, and the other polymorbid patient died on postoperative day 11 after ICA due to prolonged severe sepsis after re-do surgery for anastomotic leakage.

### Linear regression analyses

**Fig. 5:**
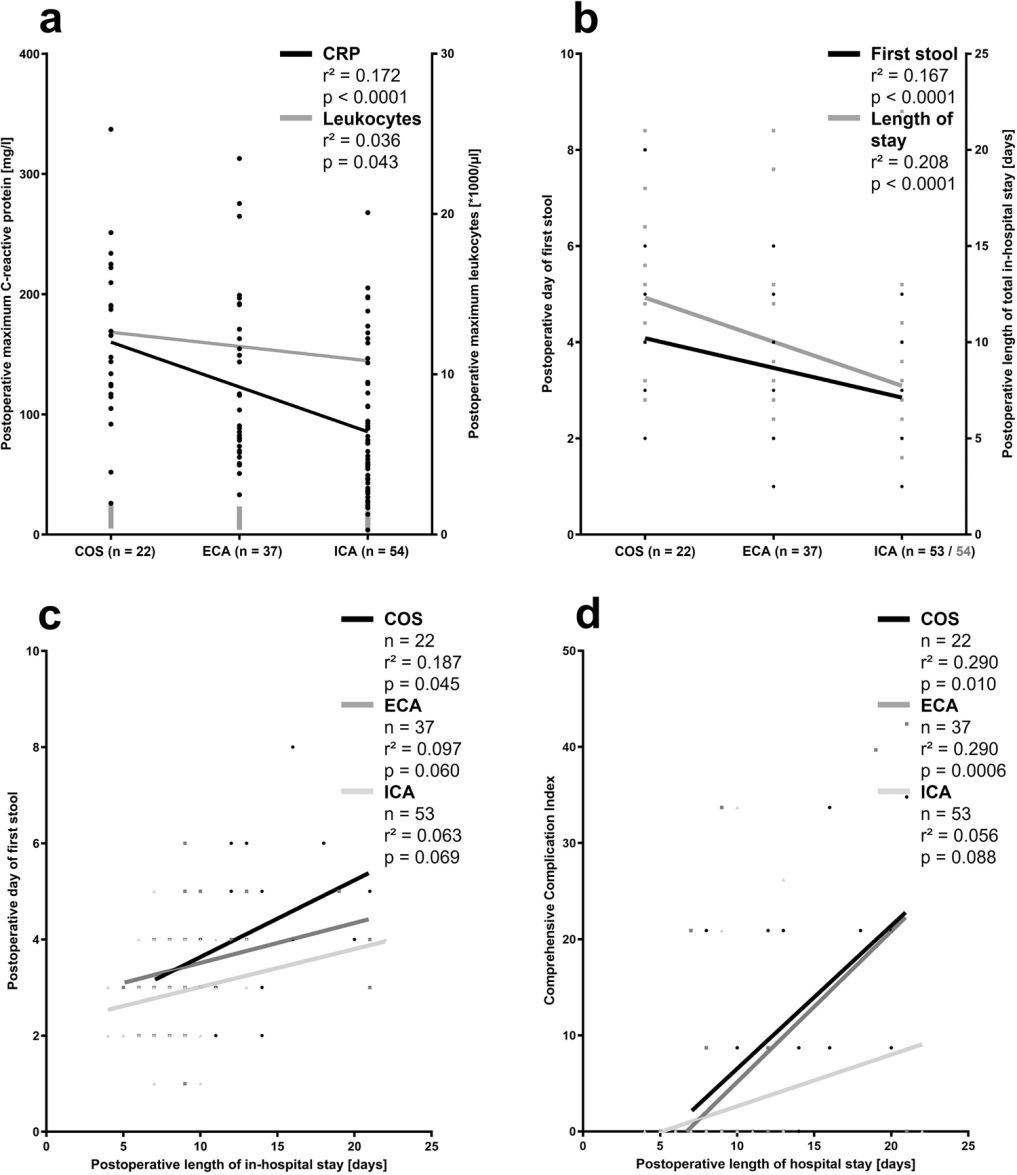
Linear regression analysis in response to the surgical approach. **a** Linear regression of highest C-reactive protein values in peripheral blood during postoperative days 1–3 (left *Y*-axis, black line and dots) and highest leukocyte counts in peripheral blood during postoperative days 1–3 (right *Y*-axis, grey line and dots) against the surgical approach. **b** Linear regression of postoperative bowel dysfunction, i.e. duration until first postoperative stool (left *Y*-axis, black line and dots) and postoperative length of total in-hospital stay (right *Y*-axis, grey line and dots) against the surgical approach. **c** and **d** Linear regression of the length of postoperative total in-hospital stay against postoperative day of first stool and comprehensive complication index differentiated by the three surgical approaches. *COS* conventional open surgery; *ECA* hybrid minimally invasive, robotic-assisted right colectomy with extracorporal hand-sewn anastomosis; *ICA* total minimally invasive, robotic right colectomy with intracorporal hand-sewn anastomosis

Linear regression analyses basically confirm the results from group comparisons. The postoperative elevation of CRP values and leukocyte counts, the postoperative day of first stool, and the length of postoperative in-hospital stay correlate significantly with the surgical approach (i.e., COS > ECA > ICA; Fig. 5a and b).

**Fig. 6:**
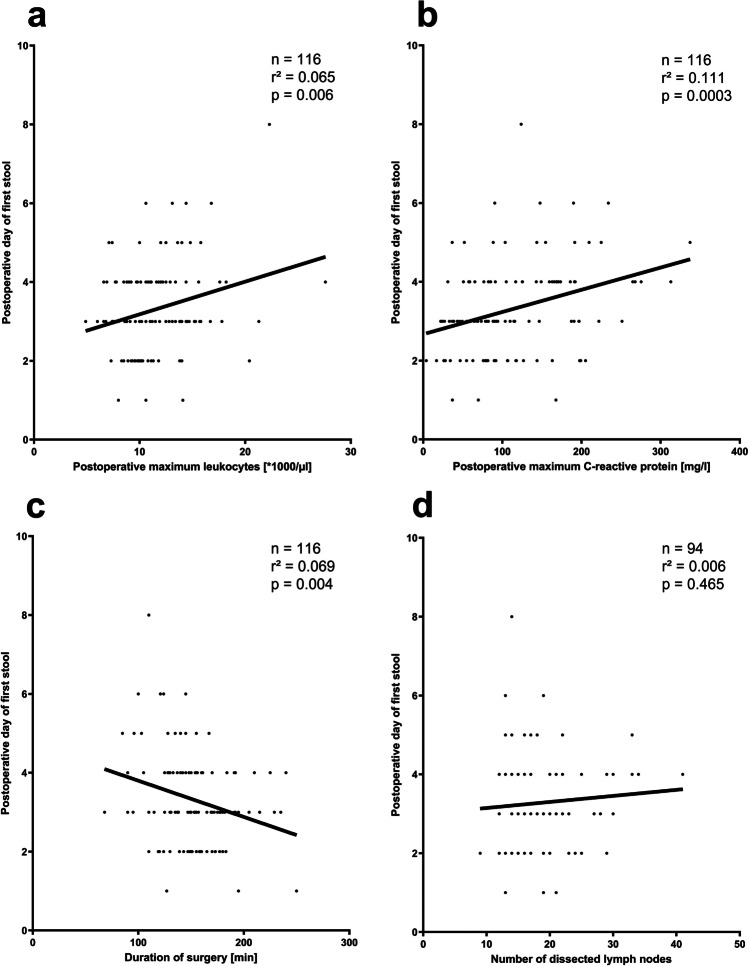
Evaluation of factors that might influence on postoperative bowel dysfunction independently from the surgical approach by linear regression analysis

Further factors were evaluated that might influence on postoperative bowel dysfunction, independently from the surgical approach. The day of postoperative first stool was dependent on the height of postoperative inflammatory markers, but not on the duration of surgery or numbers of retrieved lymph nodes (Fig. 6). Consequently, a longer duration of postoperative bowel dysfunction indicated by first postoperative stool as well as postoperative complications resulted in a longer postoperative in-hospital stay after RC. This effect was mostly obvious and significant in the COS group (Fig. 5c and d).

## Discussion

The present study describes beneficial effects of the total robotic approach on postoperative bowel recovery and general patient outcome during the learning curve of robotic RC with CME. Since implementation of the robotic surgery program, the institution had been evolved to a nation-wide benchmark center for robotic colorectal surgery [36]. However, we came from conventional open surgery as the generally accepted standard procedure and went directly to robotic surgery for oncologic RC without taking the detour via conventional laparoscopy. Hence, a comparison of conventional laparoscopy directly with robotic surgery was not performed herein, which might be a weak limitation of the present study. However, as our experience and data from the recent literature proof as well as nationwide research in medical care show, robotic surgery allows consistent results even for surgeons with no prior experience in minimally invasive, conventional laparoscopic surgery after adequate and structured training [4–7, 9, 11, 16, 37]. Although non-inferiority was proven by randomized trials early in 2005 and 2007 for laparoscopic compared with conventional open colorectal resections for cancer, conventional laparoscopic approaches did not reach general acceptance in many European countries [4–7, 9, 37]. Especially the rate of laparoscopically performed right colectomies remains low [9] with an overall increasing trend towards robotic surgery [11, 16]. This reflects the situation in many surgical departments, as ours: the decision is made between COS or robotic surgery, but conventional laparoscopy is not considered for oncologic RC. Thereby, the literature reveals for patients after robotic surgery some impactful advantages with respect to relevant clinical outcome parameters moreover laparoscopy [11, 16, 24, 31], including higher lymph node yield during CME and shorter intracorporal hand-sewn anastomosis time due to improvements in ergonomics, handling, and visualization through the robot [8, 12, 31, 38].

Procedure times seem to be an aspect of economic disadvantages of robotic surgery. Although the literature proofs shorter time for intracorporal suturing of the anastomosis during robotic surgery [12, 38], the duration of the total robotic procedure is — in contrast to the data of the present ICA group — supposed being longer compared with conventional laparoscopy and COS [11, 39, 40]. But, there is currently no standardized definition available which data should be recorded for the operation time of robotic surgery and most publications on robotics do not discriminate between port setup time, time-consuming docking, robot time, or assisted time, which includes creation and closure of the minilaparotomy, extraction of the specimen, or different steps of surgery, for example, the time for extracorporal anastomosis as well [8, 41]. Nevertheless, slightly longer operation times in comparison with standard COS (*p* = 0.044) were only observed in the present study during the initial ECA phase, immediately after introduction of robotic surgery to oncological RC, but not thereafter. Though the operative time was defined in the present study as the continuum from the first incision to complete skin closure and consecutively includes all the previously mentioned surgical steps. Hence, these quite short operation times especially after these initial (ECA) cases might be an effect of the strong two-step learning curve setting described in here. Increases in surgeon experiences and team training are essential to improve performance in robotic surgery [8, 41, 42]. In the present study we started the program with the robotic-assisted approach; thereby the ileo-colic anastomosis was sewn by hand in the conventional manner through a transverse laparotomy (ECA patients). This first step allowed us to get in touch with robotics and to gain sufficient experiences with mobilization of the colon and appropriately approaching the CME, a crucial and difficult-to-perform step in minimally invasive RC [8]. Thereafter we went to the total minimally invasive approach with intracorporal hand-swen anastomosis (ICA patients), which can be safely performed by experienced robotic surgeons [3], as the next step. Although we only saw slight differences in operation times in direct group comparison between COS and ECA, learning curve analysis confirmed these results impressively: significant decrease over time during initial ECA patients, but not anymore thereafter (ECA: *r*^2^ = 0.107, *p* = 0.048; ICA: *r*^2^ = 0.002, *p* = 0.755; data not shown). However, the duration of operation times in COS patients might reflect a minor limitation of the study through the fact that in the present patient cohort open right colectomies were performed by different surgical teams, including different surgeons (*n* = 8) at different training levels. Thereby the open procedures were performed together with a well-experienced senior surgeon at least as the first assistant. In contrast, robotic surgeries were performed exclusively by two well-experienced senior surgeons in an experienced team. Nevertheless, by the decrease over time, we conclude that procedure durations approximate the standard COS approach for RC after the initial learning phase. This was the situation for the ICA cohort and, together with the low conversion to open surgery rate in ICA patients (*n* = 1/56, 1.8%), these data proved the sufficiency of our learning curve setting. However, this learning curve effect was not observed in total lymph node yield through CME over time as a global parameter for oncologic quality of surgical approaches. Both the decrease in operation time and constant lymph node yield over time indicate the efficacy of our learning curve setting, which was accordingly completed during the initial observational period of the present study [8, 14] and allows for the conclusion that the institutional development from COS via ECA to ICA had neither impaired oncologic quality during surgery nor patient safety.

The advantages concerning short-term outcomes of minimally invasive surgery over COS in colorectal surgery are obviously evident and had been extensively shown in the current literature for RC by laparoscopic approaches, whereby oncological outcomes were not inferior [1, 43]. Although operation times of minimally invasive routes were considerably longer in previous literature, beneficial effects of minimally invasive surgery for RC were also reproduced by the robotic approaches in the present study: rate of major morbidity including re-operations, postoperative bleeding, anastomotic complications, and rates of surgical site infections, reflected by longer length of postoperative hospitalization and higher rates in CCI in the COS group [10, 11, 16, 17, 30, 31, 44]. In the recent literature no differences have been shown regarding anastomotic complications, i.e., leackages and bleeding, in comparsion of different types of anastomoses (intracorporal versus extracorporal or hand-sewn versus stapling, et cetera) [3, 45–48]. Vice versa, the intraabdominal creation of the anastomosis itself, including shorter incision length [49–51] and alternatives of the extraction site, i.e., Pfannenstiel versus midline or pararectal incisions [26, 50–52], correlate with reduced rates of surgical site infections [26, 27, 31, 53] and incisional hernia [51, 54] during short-term and long-term follow-up after RC. Furthermore, a lower pain level [50], a shorter duration until normalization of bowel function including a faster food tolerance, and time to passage of first postoperative flatus and stool contribute to improved patient comfort, enhanced patient recovery, and result in shorter length of postoperative hospital stay in patients after total minimally invasive RC with intracorporal anastomosis [26, 27, 49, 50, 52, 55].

Prolonged postoperative bowel dysfunction and ileus observed in up to 30% of the patients are frequent pathologies after RC [24] and cause prolonged food intolerance, significant dyscomfort, and potential severe morbidity for the affected patients [33–35]. Time to first stool was decided as the surrogate parameter of postoperative bowel recovery in the present study, which allows for comparison with the literature; however core outcome sets for defining postoperative gastrointestinal recovery or prolonged bowel dysfunction and ileus had not been described, yet [56]. Postoperative gastrointestinal dysmotility is a physiologic and temporary reaction following abdominal surgery, but usually returns to normal function rapidly [33, 57]. The pathophysiology of prolonged postoperative bowel dysfunction is uncertainly multifactorial but currently not fully understood [58]. Although the literature previously suggests that intracorporal anastomoses are beneficial regarding the duration of postoperative bowel recovery, our data give some important insights in the pathophysiology of prolonged gastrointestinal dysfunction: neither duration of surgery nor extent of CME with central lymph node harvesting but greater extent of ileo-colic manipulation, higher trauma to the abdominal cavity, and more severe systemic inflammatory response, both might trigger opioid-based pain therapy, contribute to prolonged gastrointestinal dysfunction in the COS and ECA groups (*p* = 0.0009 and *p* = 0.020 versus ICA, respectively) [33, 57, 59]. Furthermore, regression analyses clearly demonstrate the harmful effect of gastrointestinal dysfunction on patient outcome: delayed postoperative bowel dysfunction contributes to longer postoperative in-hospital stay especially in patients who underwent COS (*r*^2^ = 0.187, *p* = 0.045) for oncologic RC. This was comparable with other severe postoperative complications. These results reveal that the ICA procedure is clinically more effective with regard to postoperative bowel recovery and length of postoperative hospitalization.

Nevertheless, higher initial and running costs, stronger investment in surgical instruments and consumables, and longer operation times lead to ongoing debate if the benefits of robotic surgery justify economic concerns, especially competing with conventional laparoscopy [1, 11, 21, 24]. Aspects, which might finally overcome initial economic burdens, include the optimization of the surgical procedure by decreasing the duration over time through surgical team formation and strong adherence to the learning curve as well as the use of more cost-effective techniques and materials (hand-sewn instead of stapler anastomosis, conventional laparoscopic instead of robotic staplers, reduction of robotic ports). However, the most powerful impact from a clinical and economic view have lower complication rates, faster recovery, and shorter length of in-hospital stay after robotic compared with both COS and laparoscopic RC [11, 16, 20, 24]. Thus, in our study robotic surgery let to a significant reduction in cost-intensive postoperative complications, duration at the intensive care unit, and length of postoperative in-hospital stay by one third from median 12 (COS) to 8 days (both robotic approaches: ECA and ICA, *p* < 0.01 each in comparison with COS). This might all together turn initial economic disadvantages into a potential benefit and increases the revenue for robotic cases.

## Conclusion

In conclusion, the results of this single-center experience demonstrate that implementation of a robotic program for right colectomies with CME is safely feasible even without experiences in conventional laparoscopic surgery in that field. Beginning the learning curve with the robotic-assisted approach and extracorporal anastomosis is approporiate; conversion to total-robotic surgery with intracorporal anastomosis after enough experiences and surgical skills had been acquired provides excellent outcome for the patients. Especially the effects of total minimally invasive robotic surgery on postoperative functional bowel recovery and wound complications positively impact on length of hospitalization and patient comfort. This might overcome economic concerns regarding robotic surgery.
